# The Combination of Diosgenin, Vitamin D, and α-Lactalbumin Normalizes the Menstrual Cycle in Women with PCOS of Phenotype D: A Pilot Clinical Study

**DOI:** 10.3390/nu17233695

**Published:** 2025-11-25

**Authors:** Michele Russo, Giuseppina Porcaro, Cesare Aragona, Gabriele Bilotta, Massimo Di Liberto, Vittorio Unfer

**Affiliations:** 1 R&D Department, Lo.Li. pharma s.r.l., 00156 Rome, Italy; 2 The Experts Group on Inositol in Basic and Clinical Research and on PCOS (EGOI-PCOS), 00156 Rome, Italy; 3 Sotherga Clinic, 20121 Milan, Italy; 4 Systems Biology Laboratory, Department of Experimental Medicine, Sapienza University, 00163 Rome, Italy; 5 Alma Res Fertility Center, 00198 Rome, Italy; 6 Complex Operating Unit of Gynecology, Obstetrics of the “Abele Ajello” Hospital, 91026 Mazara del Vallo, Italy; 7 Department of Gynecology and Obstetrics, UniCamillus–Saint Camillus International University of Health Sciences, 00131 Rome, Italy

**Keywords:** polycystic ovary syndrome (PCOS), phenotype D, diosgenin, vitamin D, α-lactalbumin, menstrual cycle regularity

## Abstract

Background: The study aimed to evaluate the efficacy of a combination of *Dioscorea villosa* (containing diosgenin), vitamin D, and α-lactalbumin, in women with Polycystic Ovary Syndrome (PCOS) phenotype D. The primary objective was to investigate improvements in menstrual cycle regularity. Methods: A total of 24 women aged 22–34 years with PCOS phenotype D received daily supplementation with 600 mg *Dioscorea villosa* (120 mg diosgenin), 100 mg α-lactalbumin, and 50 μg vitamin D for six months. Clinical and biochemical assessments, including hormonal profiling and menstrual cycle monitoring, were conducted at baseline (T0), after 3 months (T1), and after 6 months (T2). Results: The treatment led to a statistically significant improvement in menstrual cycle regularity: eumenorrhea was achieved in 50% of patients at T2, compared to 0% at baseline. Significant changes were also observed in luteinizing hormone (LH), follicle-stimulating hormone (FSH), and the LH/FSH ratio, alongside a reduction in insulin and HOMA-index at T1. No adverse events were reported. Conclusions: The combination of *Dioscorea villosa*, vitamin D, and α-lactalbumin promotes menstrual cycle regularization in women with PCOS phenotype D. The positive result suggests a beneficial role of the treatment when administered to this specific subtype of PCOS and supports the use of targeted nutraceutical therapy as an alternative to conventional treatments, especially in non-hyperandrogenic PCOS patients.

## 1. Introduction

Polycystic Ovary Syndrome (PCOS) is a common metabolic–endocrine disorder affecting individuals of reproductive age, characterized by a diverse clinical presentation and complex pathophysiology. The condition is widely recognized as one of the leading causes of infertility, metabolic dysregulation, and psychological distress in women [1]. PCOS is characterized by the presence of hyperandrogenism, ovulatory dysfunction, and polycystic ovarian morphology, the combination of which results in distinct phenotypes that reflect its multifaceted nature. These phenotypes provide insight into the heterogeneity of the disorder, offering opportunities for tailored diagnostic and therapeutic approaches.

The four primary PCOS phenotypes, as outlined by the Rotterdam Criteria [2], include the following: hyperandrogenism, ovulatory dysfunction, and polycystic ovarian morphology (phenotype A), hyperandrogenism and ovulatory dysfunction (phenotype B), hyperandrogenism and polycystic ovarian morphology (phenotype C), ovulatory dysfunction and polycystic ovarian morphology (non-hyperandrogenic PCOS, phenotype D).

The four PCOS phenotypes differ markedly in their clinical, biochemical, and metabolic profiles: hyperandrogenic phenotypes (A–C) generally present with higher serum androgens, insulin resistance, and greater cardiometabolic risk, whereas phenotype D, lacking hyperandrogenism, shows milder metabolic alterations, lower LH/FSH ratio, and better reproductive outcomes, highlighting the heterogeneity of the syndrome [3,4,5,6]. Understanding these distinctions is critical, as they influence both the manifestation of the syndrome and the management strategies needed. For instance, the presence or absence of hyperandrogenism significantly affects diagnostic approaches, while variations in metabolic risk across phenotypes necessitate tailored interventions [7].

PCOS phenotypes have different distributions depending on the regional and national data available, particularly the prevalence of the PCOS subgroup with phenotype D is highly variable around the world [3]. A distinct characteristic of patients with PCOS phenotype D is that they suffer from irregular menstrual cycles. This most typically manifests as oligomenorrhea (menstruations ≥ 35 days apart) or amenorrhea (menstruations ≥ 45 days apart and/or the absence of menstruations for three months) but may also occur as polymenorrhea (menstruations ≤ 21 days apart) [8,9].

Women classified as PCOS phenotype D exhibit a distinct clinical profile characterized by relatively preserved metabolic parameters and lower androgen levels compared with the classic hyperandrogenic phenotypes A–C. Moreover, these women with phenotype D are more prone to successful fertility procedures and exhibit lower levels of AMH compared to hyperandrogenic phenotypes [10]. Historically, diagnostic frameworks that require hyperandrogenism (NIH; AE-PCOS Society) would therefore exclude phenotype D from the PCOS diagnosis; however, recent reappraisals emphasize that phenotype D may represent a primarily ovarian-onset form of the syndrome. As such, ovulatory dysfunction and follicular arrest are strictly involved in the pathophysiology and thus argue for its proper recognition as PCOS despite the absence of overt hyperandrogenism [11]. This perspective suggests that phenotype D differs in etiology and reproductive implications from hyperandrogenic phenotypes and may require phenotype-tailored diagnostic and therapeutic approaches rather than categorical exclusion.

To date, no specific medications exist to treat women with PCOS phenotype D. Typically, these patients are prescribed combined oral contraceptive pills (OCPs) to restore physiological menstrual cyclicity; however, several studies have suggested that OCP prescription may worsen cardiometabolic parameters, so that they cannot always be recommended [12]. Moreover, contraceptive therapy must be suspended when seeking pregnancy.

Supplements, or nutraceutical approaches, may offer a valid alternative to OCP therapy in these patients. One such natural molecule that has drawn interest is diosgenin, a natural steroid sapogenin contained in the plant family of Dioscoreacee, particularly in the Rhizoma of *Dioscorea villosa*, which has been used in traditional Chinese medicine for the regulation of menstrual cycles and the amelioration of menopausal symptoms [13]. The precise mechanism of action of diosgenin is unknown; therefore, a recent study sought to use network pharmacology and molecular docking to systematically predict its potential molecular targets and associated signaling pathways.

In this study, bioactive compounds from *Dioscorea alata* L., including diosgenin, were demonstrated to interact with key molecular targets of menstrual disorders, known to modulate pathways involved in ovarian follicle formation, hormone regulation, estrogen receptor signaling, and the stress-activated MAP kinase pathway [14]. Diosgenin can act by sustaining the luteal phase in those patients characterized by altered menstrual cycles, as demonstrated in a rat model where diosgenin increases the number of corpora lutea [15]. These data suggest the possible use of this compound to improve the ovulation rate, particularly in patients with conditions like PCOS, who frequently suffer from ovulatory disorders.

Another molecule of interest for the regulation of menstrual cyclicity is vitamin D. Vitamin D can be classified as a secosteroid, a chemical class of compounds that are defined by a steroid-like structure in which a ring cleavage between carbon atoms has occurred [16]. It is for this reason that vitamin D has previously been described as a “progesterone-like” molecule and has been found to synergize with progesterone during pregnancy [17]. Vitamin D plays an important role in the modulation of the anti-Mullerian hormone (AMH) in addition to follicle-stimulating hormone (FSH) within the ovary, as discovered through initial studies in hens [18]. This work laid the foundations for subsequent studies in humans, where AMH is known to be involved in follicular genesis, with excessive levels worsening ovarian function, increasing the risk of oligo or amenorrhea [19]. Of note, women with PCOS have been shown to have significantly higher levels of AMH in comparison with healthy populations [20], and vitamin D may be helpful. Indeed, it inhibits the expression of AMH receptor (AMHR) and FSH receptor (FSHR), thus facilitating the correct follicle maturation [21]. Moreover, vitamin D increases the mRNA levels of 3beta-hydroxysteroid dehydrogenase (3β-HSD), a steroidogenic enzyme involved in the production and release of progesterone, which is fundamental for the luteal phase of the menstrual cycle.

Together with diosgenin and vitamin D, the globular whey protein α-lactalbumin has been increasingly investigated as an important supplement in the treatment of PCOS, considering that the syndrome seems strongly connected with gastrointestinal dysbiosis [22]. In fact, due to its prebiotic activity, α-lactalbumin supplementation can restore gut homeostasis and reduce body weight (and BMI), in addition to improving glycaemia/insulin sensitivity, triglycerides, and low-density lipoprotein levels [23]. Although these metabolic comorbidities are more frequently associated with hyperandrogenic PCOS, the study of α-diversity also demonstrates an important alteration of the gut microbiota in women with PCOS phenotype D; therefore, α-lactalbumin may also find a suitable application in these patients [24].

Considering the potential beneficial effect of these three natural molecules, this paper describes the effect of a novel treatment specifically formulated for patients with PCOS phenotype D. By delineating distinctions between phenotypes, we seek to enhance the understanding of PCOS heterogeneity and its implications for personalized medicine, ultimately contributing to improved outcomes for patients.

## 2. Materials and Methods

In the present prospective single-arm pilot study, we enrolled 24 women (aged 22–34) with a diagnosis of PCOS with phenotype D according to the Rotterdam Criteria (ESHRE Guidelines for PCOS 2023). As such, we included women characterized by menstrual cycle disorders, such as oligo-/amenorrhea, and evidence of polycystic ovarian morphology. Since participants presented with oligo- or amenorrhea and were not seeking pregnancy, ovulatory dysfunction was assessed based on menstrual cycle irregularities, as commonly reported in the literature. Progesterone levels were not measured to avoid potential misinterpretation, given the asynchronous or irregular timing of the mid-luteal phase in these patients.

Exclusion criteria were as follows: Free Androgen Index(FAI) > 4.5, presence of insulin resistance assessed with Homeostasis Model Assessment (HOMA-index) > 2.5, and use of oral contraceptives or products containing *Dioscorea villosa* extract, and/or vitamin D, and/or α-lactalbumin 6 months prior to the start of the study.

Women with differential diagnoses of Cushing’s disease, non-classical 21-hydroxylase deficiency, adrenal hyperplasia, hyperprolactinemia, and androgen-secreting tumors were excluded from participation. Also, women with menopause and perimenopause, ongoing and seeking pregnancy, breastfeeding, substance abuse, acute illness, diagnosis of diabetes, recent history or presence of endometrial hyperplasia or carcinomas, and/or other neoplasms were excluded from the study.

The Internal Review Board of the ethical committee of the Alma Res Clinic approved the study (record number: 011/2022, date 15 December 2022), which was registered on clinicaltrials.gov (ID: NCT06639698) and conducted according to the ethical principles of the Declaration of Helsinki. Participants were enrolled at Alma Res Clinic and then referred to the following medical centers to receive treatment and follow-up: Agunco Medical Center (Rome, Italy) and Department of Gynecology and Obstetrics, Women’s Health Centre (Terni, Italy) between October 2024 and June 2025. All participants signed a consent form for participation in the study.

The patients who met the inclusion criteria were treated daily with 600 mg *Dioscorea villosa* (containing 20% diosgenin—120 mg), 100 mg α-lactalbumin, and 50 μg vitamin D (Inofolic pHD commercialized by Lo.Li. pharma s.r.l) for 6 months.

Blood tests and anamnestic data were collected at baseline (T0), after 3 months of treatment (T1), and after 6 months of treatment (T2). These included Body Mass Index (BMI), levels of total testosterone, sex hormone-binding globulin (SHBG), Free Androgen Index (FAI), insulin, HOMA-index, luteinizing hormone (LH), FSH, and LH/FSH ratio. We recorded the menstrual cycle regularity at each timepoint. Amenorrhea was identified as absent menstrual cycles for >90 days, oligomenorrhea was defined as two subsequent menstrual cycles between 35 and 90 days apart, polymenorrhea was defined as frequent menstrual cycles (<21 days apart), and regular menstrual cycle or eumenorrhea was identified with menstrual cycles between 21 and 35 days apart.

The statistical analysis was conducted in the first line with the Shapiro–Wilk Test to assess the normal distribution of data referring to each parameter evaluated in the study. Subsequently, we performed a Wilkoxon Signed-Rank Test to compare the different timepoints for each parameter and the symmetry test to compare the distribution of the different types of menstrual cycle alteration at each timepoint. Since no previous study aimed to test the effects of the described formulation in the subset of women with PCOS phenotype D, we could not calculate or estimate a sample size, and the present study should be considered as a pilot investigation.

## 3. Results

The baseline characteristics of the patients enrolled in the study are reported in Table 1, along with T1 and T2 values. Median values are reported for all parameters, with the 25th and the 75th percentiles in square brackets.

The population selected has a median age of 26.5 years, and the women are not obese, with a median weight of 61.50 kg and a median BMI of 22.4. Patients exhibited a median value of FAI equal to 3.55 (3.31–4.10), which indicates the absence of biochemical hyperandrogenism in these patients, according to the cut-off value of 4.5. We observed a significant variation in FAI in both the comparison of T2 with T0 and T2 with T1; however, the values remained below 4.5 for the entire treatment period. Endorsement of FAI reliability published in ESHRE updated guidelines 2023 [2] and cut-off value of 4.5 reported by van Keizerswaard et al., 2022 [25].

A significant variation is observed for testosterone comparing T1 vs. T0 and for SHBG comparing T1 vs. T0 and T2 vs. T1. We also reported a significant reduction in HOMA-index when comparing T1 with baseline, and T2 with T1.

Gonadotropins were also investigated in the study, highlighting a significant reduction in LH comparing T2 to baseline and T2 with T1, and a significant decrease in FSH for the comparisons of T2 vs. T0. Also, a significant decrease resulted in the LH/FSH ratio when T2 is compared to T1. As a cut-off for suspect biochemical hyperandrogenism, we referred to values > 1.5 according to Wiser et al. [26].

Weight, and therefore BMI, did not exhibit any significant variation over the treatment period.

**Figure 1:**
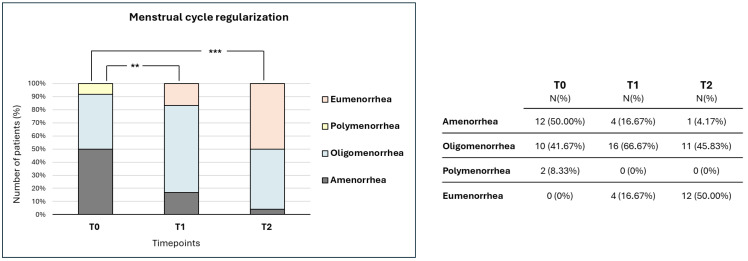
Menstrual cycle regularization over the treatment period. The chart reports the distribution of the menstrual cycle alteration over the treatment period. Data are reported as percentage values at three different timepoints: T0 (baseline), T1 (3 months of treatment), and T2 (6 months of treatment). *p*-values are described as follows: ** *p* < 0.01, and *** *p* < 0.001. Values in the table indicate the number of patients and the percentage of reference at each timepoint.

As shown in Figure 1, the menstrual cycle regularity significantly changed already after 3 months of treatment; after 6 months, these differences were even more significant. Indeed, at baseline, we observed 50% of women with amenorrhea, 41.67% of women with oligomenorrhea, 8.33% of women with polymenorrhea, and no patient with eumenorrhea. At T1, we recorded 16.67% of women with amenorrhea, 66.67% of women with oligomenorrhea, no patient with polymenorrhea, and 16.67% of women with eumenorrhea. At T2, we observed 4.17% of women with amenorrhea, 45.83% of women with oligomenorrhea, no patient with polymenorrhea, and 50% of women with eumenorrhea. Finally, none of the participants reported any adverse events during the treatment period.

## 4. Discussion

In this pilot study, we found that after 6 months of supplementation with *Dioscorea villosa*, vitamin D, and α-lactalbumin, 50% of patients with PCOS of phenotype D recovered a regular menstrual cycle.

Interestingly, at baseline, 50% of the study group were affected by amenorrhea, 41.67% were affected by oligomenorrhea, and 8.23% exhibited polymenorrhea. Of note, none of the participants in the study showed a regular menstrual cycle. After 3 months of treatment, an improvement in the menstrual cycle regularity is observed, and the menstrual cycle normalization is further enhanced, considering that 50% of the patients achieved a regular menstrual cycle after 6 months of treatment. This result suggests a beneficial role of the treatment for this specific subtype of PCOS patients.

The importance of distinguishing PCOS phenotypes to improve the diagnosis of the syndrome has already been discussed by our group in previous publications [11,27]. In several clinical studies published to date, this aspect seems highly neglected, so women with PCOS are enrolled in studies and clinical trials as a single study group without stratification or separation by phenotypes. This will influence the perception of the effect of a specific treatment, since the study includes patients who may not benefit from the intervention and who need a different therapeutic approach altogether. In this context, phenotype D seems particularly interesting as it represents the only non-hyperandrogenic PCOS phenotype, characterized by oligo/-anovulation combined with the presence of polycystic ovarian morphology.

Indeed, with respect to the hyperandrogenic phenotypes, women with PCOS phenotype D are less exposed to metabolic alterations, increased HOMA, deregulated lipid profile, and reduced insulin sensitivity [28,29,30,31]. For this reason, these patients do not benefit as much as hyperandrogenic patients from therapies with insulin sensitizers, such as metformin or myo-Inositol [32], and they need a different intervention. The therapeutic rationale should consider that, probably in phenotypes A, B, and C, the metabolic alteration drives the hyperandrogenism [7], suggesting that these patients likely exhibit an endocrine–metabolic syndrome rather than a specific ovarian pathology [27]. On the other hand, women with PCOS phenotype D exhibit normal circulating androgens, particularly testosterone, and a low rate of dysmetabolism compared to hyperandrogenic phenotypes [33]. Frequently, these women also have low androstenedione and normal levels of SHBG, which is typically suppressed in the hyperandrogenic phenotypes, so that phenotype D patients are characterized by low levels of FAI in comparison to hyperandrogenic phenotypes [34]. As a consequence, women with PCOS phenotype D are less exposed to clinical manifestations of hyperandrogenism, including hirsutism, acne, and alopecia [35], and they necessitate alternative treatments with respect to those used for women with hyperandrogenic PCOS phenotypes.

On this basis, and considering the presence of altered ovarian functions, the EGOI-PCOS group recently hypothesized a separate and unique etiopathogenesis for phenotype D of PCOS.

In particular, they speculated that a potential underlying mechanism for the ovarian disturbances observed in this specific phenotype may be represented by elevated IGF-1 levels [11]. IGF-1 is a growth factor that is known to be vital for numerous biological processes, including ovarian function [36]. In an ex vivo study, Dai et al. observed that, while low doses of IGF-1 improve the growth of murine ovarian follicles, the administration of higher doses (50 ng/mL) resulted in the arrest of follicular growth [37].

As it pertains to this work, a link between IGF-1 and diosgenin was reported by Mu et al., who investigated the antiproliferative effect of diosgenin in human thyrocytes to evaluate its potential in treating goiters associated with Graves’ disease [38]. It has been previously demonstrated that IGF-1 plays a role in goiter growth, with its signaling thought to stimulate proliferation and impair apoptotic functions [39]. In the study, human thyrocytes were stimulated with IGF-1, causing significantly enhanced proliferation, and the administration of diosgenin resulted in a significant dose-dependent reduction in cell viability. The researchers confirmed that apoptosis was involved, as they observed that the administration of diosgenin activated the caspase cascade, triggering both FAS-related and mitochondrial apoptotic pathways. This suggests that diosgenin may be able to counteract the abnormal activity of IGF-1.

In addition, diosgenin may sustain the production of IGF-1 binding protein (IGFBP-1), thus quenching the excessive biological activity of IGF-1 [40]. Indeed, diosgenin first garnered attention from the endocrinological and gynecological medical community as it is routinely used in the laboratory synthesis of progesterone [13] and has been used in traditional medicine for the treatment of menopause, with the hypothesis that diosgenin would be converted into progesterone once ingested. However, this conversion has only been demonstrated in vitro, and, to date, there is no evidence that this may occur in the human body [41]. Likewise, vitamin D is a “progesterone-like” molecule with a steroid-like structure [16], able to synergize with progesterone during pregnancy by stimulating anti-inflammatory pathways and by inhibiting proinflammatory pathways [17,42].

Of note, IGFBP-1 is up-regulated under progesterone input and promotes the decidualization of the endometrial stromal cell via alpha5beta1 integrin sustained by progesterone [43,44]. Indeed, progesterone-responsive elements have been mapped in the promoter of the human IGFBP-1 gene [45]. In this scenario, it might be possible that diosgenin and vitamin D may synergize in increasing IGFBP-1 expression and consequently inhibiting the deregulated signaling of IGF-1. Also, they may enhance progesterone levels, which are essential for allowing the beginning of the luteal phase, and to restore a physiological ovulation process. This may explain the beneficial effect on the menstrual cycle regularity observed in the study and the importance of diosgenin associated with vitamin D in this pathological context. However, to date, we lack sufficient data to confirm this hypothesis, even if it does seem to be an interesting topic for future research, with its focus on eventual diosgenin modulation of IGF-1/IGFBP-1 levels in these patients.

Diosgenin is a phytoprogestin molecule that can be retrieved in root, leaf, and rhizome extracts of *Dioscorea villosa* [46,47,48]. Several authors suggest a potential beneficial role of diosgenin for the entire reproductive system of women [49], thus supporting a possible use of this molecule in PCOS. Even though randomized clinical trials are lacking, preclinical in vivo data, particularly in mouse models, and some human trials with *Dioscorea* preparations, suggest a biologically plausible role for diosgenin in improving ovarian function and menstrual parameters. Shen et al. (2017) [50] showed that diosgenin increased follicle counts and serum AMH in mice, while in rats, diosgenin treatment increases the number of corpora lutea, thus positively sustaining the ovulation rate [15].

*Dioscorea esculenta* supplementation in humans reduced PGE_2_/COX-2 and menstrual pain [51], and letrozole induced PCOS rat models treated with Dioscin, the saccharide derivative of diosgenin, demonstrating an increase in progesterone levels after Dioscin administration [52].

Our hypothesis is that diosgenin, as well as vitamin D, may act synergistically as progesterone-like molecules, thus contributing to the restoration of a regular menstrual cycle in patients with PCOS phenotype D.

Vitamin D also plays a pivotal specific role in follicle development, sustaining the entire process in both gonadotropin-independent and gonadotropin-dependent phases [53]. During follicle maturation, vitamin D receptors (VDRs) start being expressed, thus facilitating oocyte meiosis and granulosa cell proliferation. Subsequently, vitamin D enhances the secretion of estrogens and progesterone from the follicular cells, modulates AMH and FSH signaling, and promotes ovulation.

Considering the above, it is not surprising that vitamin D deficiency has been demonstrated to be significantly correlated with menstrual cycle disorders. Specifically, Łagowska reported that women who did not reach recommended levels of vitamin D (30 ng/mL) were almost five times more likely to have menstrual cycle disorders [54].

The whey protein α-lactalbumin seems to counteract gut dysbiosis, which has been increasingly connected with PCOS [55,56,57] as it is related to metabolic factors such as obesity, insulin resistance, and intestinal inflammation [58]. Due to its prebiotic effect, α-lactalbumin can improve the α-diversity of microbiota in the gut, thus favoring the growth of health-promoting bacteria yet limiting the proliferation of potential pathogens [55]. Particularly, the data demonstrate that oral administration of α-lactalbumin for 30 days in women with PCOS can partially restore a healthy gut and vaginal microbiota by promoting the growth of beneficial genera like *Bifidobacterium* and *Lactobacillus* [55]. According to the DOGMA (Dysbiosis of Gut Microbiota) theory published by Tremellen and colleagues in 2012, an altered condition of gut microbiota may contribute to PCOS development [56]. In this study with 163 women, the authors observed that PCOS patients exhibited reduced gut microbiome diversity (α diversity) compared to healthy women. Furthermore, higher total testosterone levels and clinical signs of hyperandrogenism (such as hirsutism) were negatively correlated with microbial diversity, suggesting that androgens may influence the composition of the gut microbiota in women with PCOS [59]. As such, α-lactalbumin may contribute to ameliorating gut homeostasis, which is also from patients with PCOS phenotype D, even if with a lower severity with respect to the hyperandrogenic phenotypes [24]. In addition, α-lactalbumin can act as an effective carrier for vitamin D, improving its intestinal absorption [60]. Accordingly, the presence of α-lactalbumin in the formulation may be useful for its prebiotic activity and by enhancing vitamin D and diosgenin function.

In the present study, we also made unexpected observations concerning the variation in LH/FSH ratio and HOMA-index following the treatment with diosgenin, vitamin D, and α-lactalbumin. Even though all the changes that we recorded stay within the range of physiological values and may have no clinical relevance, the fact that significance is reached in some cases cannot be overlooked a priori.

Unlike the other phenotypes, women with PCOS phenotype D seldom exhibit an altered LH/FSH ratio, which is more generally associated with hyperandrogenic phenotypes together with an altered LH pulse frequency [61]. In our study, even if the baseline value of the LH/FSH ratio lies within the physiological range, we observed a reduction associated with the treatment, leading to values below 1 at T2. One likely explanation for this effect is that diosgenin and vitamin D inhibit LH signaling in the same manner as progesterone, allowing a physiological priming of the luteal phase and supporting the ovulation process [62].

Lastly, we reported a significant variation in the HOMA-index in treated patients, even if these women were not insulin resistant. We have no information on the molecular mechanisms that can explain this observation, given that such an investigation falls outside the scope of the present study, but in vitro experiments are currently ongoing to shed light on whether the combination of the three active molecules directly impacts insulin and LH pathways. However, we should keep in mind that a limitation of the present study is that we have not controlled the patients’ lifestyles over the treatment period, and diet and physical exercise may have an influence on the values observed. Furthermore, we did not investigate lipid and metabolic markers that could have been useful to have a better understanding of the pathological mechanisms involved in these patients. In addition, considering the quantification of the vitamin D serum levels may also be useful to assess seasonal variation and better clarify the advantages of this molecule when associated with diosgenin and α-lactalbumin. As such, a larger clinical trial with a control group included will be necessary to validate these preliminary data.

## 5. Conclusions

PCOS is a complex and heterogeneous syndrome, and patients affected present with different clinical conditions, so specific treatment should ideally be recommended for each subgroup of patients, defined as phenotypes. The present study highlights the benefits of treating patients with PCOS phenotype D (as per Rotterdam Criteria) with the association of *Dioscorea villosa*, vitamin D, and α-lactalbumin. This approach yielded a significant regularization of the menstrual cycle, and an unexpected reduction in the LH/FSH ratio, FAI, and HOMA-IR index, though within the range of physiological values. Even though all the molecular mechanisms through which these compounds act in the ovary have not been fully explained, this study suggests a potential novel approach for women with PCOS phenotype D, as they may not benefit from the canonical therapies commonly recommended to PCOS patients, such as insulin sensitizers. Also, natural molecules offer an interesting alternative treatment to OCPs, which are routinely utilized in PCOS, without any unwanted contraceptive or known adverse effects. The present manuscript supports the importance of an appropriate diagnosis and an accurate distinction of PCOS patients to evaluate tailored and effective therapies. However, further studies are still needed to confirm these preliminary findings and to better identify the single role of these molecules in the ovary and how they synergize in the pathological context of PCOS with phenotype D.

## Figures and Tables

**Table 1:** Values of the parameters recorded at baseline (T0), after 3 months (T1), and after 6 months (T2).

	Baseline Values (T0)	Values at 3 Months (T1)	Values at 6 Months (T2)	*p*-Value T1 vs. T0	*p*-Value T2 vs. T0	*p*-Value T2 vs. T1	Effect Size T1 vs. T0	Effect Size T2 vs. T0	Effect Size T2 vs. T1
Age	26.5 [24–30.25]								
Weight (kg)	61.5 [57.75–65]	62 [57.75–65]	61 [58–64.25]	0.8779	0.6719	0.4673	0.01	0.01	0.03
BMI (kg/m^2^)	22.39 [20.88–23.76]	22.5 [21.38–23.58]	22.45 [20.88–23.53]	0.5879	0.7969	0.4263	0.03	0.01	0.04
Testosterone (nmol/L)	2.00 [1.59–2.22]	1.81 [1.6–2.1]	1.95 [1.55–2.22]	0.0071	0.1289	0.3493	0.262	0.156	0.098
SHBG (nmol/L)	61.50 [52–75.25]	53.25 [46–57.63]	63 [52.68–78.75]	0.0155	0.2866	0.0011	0.21	0.111	0.308
FAI	3.55 [3.31–4.1]	3.67 [3.02–4.04]	3.09 [2.29–3.63]	0.598	0.0366	0.0428	0.055	0.21	0.204
HOMA	1.64 [1.20–2.06]	1.90 [1.66–2.07]	1.58 [1.12–1.76]	0.0312	0.3566	0.0004	0.216	0.096	0.328
LH (mU/ml)	8.10 [2.78–11.4]	6.45 [5.78–7.33]	3.1 [2.8–4.13]	0.3566	0.0057	<0.0001	0.096	0.268	0.407
FSH (mU/ml)	4.25 [3.08–5.75]	4.2 [3.78–4.5]	3.55 [3–4.3]	0.3416	0.0236	0.1326	0.099	0.212	0.155
LH/FSH	1.26 [0.86–2.13]	1.67 [1.39–1.97]	0.93 [0.82–1.15]	0.2617	0.0616	<0.0001	0.117	0.19	0.402

Body Mass Index (BMI), levels of total testosterone, sex hormone-binding globulin (SHBG), Free Androgen Index (FAI), insulin, HOMA-index, luteinizing hormone (LH), and follicle-stimulating hormone (FSH).

## Data Availability

Data is contained within the article. The complete data repository presented in this study is available on reasonable request from the corresponding author due to privacy restrictions.
